# What are the priority welfare issues facing parrots in captivity? A modified Delphi approach to establish expert consensus

**DOI:** 10.1017/awf.2024.57

**Published:** 2024-11-20

**Authors:** Rhianne Chalmers, Jonathan Cooper, Beth Ventura

**Affiliations:** Department of Life Sciences, University of Lincoln, Joseph Banks Building, Green Lane, Lincoln LN6 7TS, UK

**Keywords:** Animal welfare, captivity, environmental enrichment, housing, nutrition, parrot behaviour

## Abstract

Parrots (*Psittaciformes*) are widely kept in captivity, yet their welfare is under-researched in comparison to other captive species. This study aimed to determine key welfare issues affecting parrots through a modified Delphi approach. Twenty-eight welfare issues were first compiled via a preliminary literature review. Parrot welfare experts and sector professionals (n = 26) were then recruited to participate in an online survey to rank the identified welfare issues on a six-point scale according to severity, duration and prevalence of each issue. Participants could provide commentary on their ranking and propose additional welfare issues of concern. Items with a mean score of 4 or above progressed to a second survey, where participants (n = 14) indicated whether they agreed or disagreed with the current ranking of the welfare issue. Finally, two online workshops were held, where participants (n = 7) discussed the rankings from the second survey and sought to establish a consensus on the top ten welfare issues in each category and overall. Six of the seven final participants agreed with the final rankings, achieving a consensus rate of 86%. The top welfare issues overall were lack of owner knowledge and support; social isolation; housing; environmental opportunity to express behaviours; nutrition; development of normal behaviour; lack of a ‘life plan’ for birds; abnormal behaviours; lack of parrot-specific veterinary training; and insufficient application and enforcement of legislation. It is hoped that identification and recognition of these priority areas will be useful in directing future efforts in research, owner and veterinary education, and policy initiatives to improve parrot welfare.

## Introduction

Parrots (order *Psittaciformes*) are popular pets, with worldwide populations estimated at 50 million (see Mellor *et al.* 2021). In addition to their role as companion animals, parrots are widely kept in zoos, rescue centres and, to a lesser extent, laboratories (Frynta *et al.* 2010). However, captivity imposes risks to parrot welfare, as management and housing conditions can fail to meet parrots’ ecological and ethological needs, resulting in health and behaviour problems (for a review, see Baukhagen & Engell 2022). Compounding this situation is the limited research on companion bird (including parrot) welfare in comparison to other pet (mammalian) species (Arluke *et al.* 2015, see Burmeister *et al.* 2022).

Where welfare concerns have been raised for captive animals and for underrepresented species in research in particular, Delphi studies have been utilised to solicit expert opinion and prioritise welfare issues to direct education and research efforts (see Rioja-Lang *et al.* 2019a,b; Pearson *et al.* 2021). The Delphi methodology, originating from studies carried out by the RAND Corporation in the 1950s (Okoli & Pawlowski 2004), presents participants with the opportunity to respond anonymously to a research question, allowing the presentation of unbiased ideas and potentially leading to consensus (Rowe & Wright 2001). Delphi approaches typically consist of several phases in which experts are presented with questions refined from previous phases. Based upon previous studies (Henderson & Rubin 2012; Slade *et al.* 2014; Vogel *et al.* 2019), a 70% agreement rate is accepted for a valid consensus.

This study used a modified Delphi expert consultation to: (1) identify current welfare issues affecting parrots in captivity, incorporating the perspectives of animal welfare experts and parrot sector professionals with relevant expertise; and (2) achieve expert consensus on priority welfare issues to guide future research and educational initiatives.

## Materials and methods

### Ethical status

This study received a favourable ethical opinion from the University of Lincoln’s Ethics Committee (2023_11911). Informed consent was obtained from all participants, who could voluntarily withdraw from the study at any time.

### Study framing

In recognition of competing conceptions of animal welfare in the literature (see Fraser *et al.* 1997), we did not prescribe a single definition of animal welfare for study participants, instead allowing them to approach the study tasks from their own frames of reference. However, this study recognises that welfare issues can be conceived of both in terms of the inputs to the animal (e.g. husbandry, environment, and management factors) as well as outputs (i.e. animal-level outcomes indicating how the animal responds), and this is reflected in the lists of generated welfare issues. The term ‘parrots’ referred to the known extant 398 species of parrot or ‘psittacine’ in the order *Psittaciformes* (Wright *et al.* 2008) housed in captive settings, including private homes or collections as pets, breeding or research facilities, rescue and/or rehabilitation centres, and zoological collections for conservation and display.

### Survey population and recruitment

#### Inclusion criteria

Parrot experts and sector professionals were recruited as those with a breadth of knowledge and experience with parrots as a result of having either been engaged as avian veterinarians, behaviourists or researchers working in parrot welfare (e.g. parrot welfare, cognition, and behaviour scientists), or who had at least three years’ experience working in the parrot sector (e.g. in housing, care, selling, breeding, rehabilitation, rehoming, education or law enforcement of captive parrots). All participants were required to be over 18 years of age.

#### Participant recruitment

Delphi studies have been conducted with participant pools ranging from seven to 100 experts (Iqbal & Pipon-Young 2009). As a rule of thumb, Clayton (1997) suggested between 5–30 individuals depending on whether groups are homogeneous or heterogeneous. In our study, the expert population was considered homogeneous concerning the topic but heterogeneous in terms of professional roles, so we sought to recruit around 30 experts. This target also considered potential participant drop-out, a recognised limitation of the Delphi methodology, thereby expediting chances for a suitable sample size (Donohoe & Needham 2009).

Recruitment of experts and sector professionals began by building a list of contacts of experts and sector professionals in academia, research, veterinarian, zoo, and rescue settings known to members of the research team. Ninety-three individuals were identified globally and contacted via email or social media with an invitation to participate in the study. Snowball sampling was also employed whereby contacted individuals were invited to circulate the study invitation to others in their network. Of those contacted, 26 individuals consented to join the study.

### Study phases

This study consisted of three iterative phases: preliminary review of the literature to inform survey development; online priority identification surveys; and online workshops.

#### Preliminary review of the literature

Whilst previous Delphi studies have relied upon participants to identify welfare issues in early rounds (e.g. via online discussion boards; Rioja-Lang *et al.* 2019a,b), we chose to adopt the approach used by Keeling *et al.* (2021) and Whittaker *et al.* (2021), where an informal review of the scientific literature was performed to establish a preliminary list of captive parrot welfare issues. Google Scholar, PubMed, Web of Science, and the University of Lincoln’s online research repository were searched using keywords of ‘welfare’, ‘nutrition’, ‘health’, ‘environment’, and ‘behaviour’ in connection with the words, ‘parrot’, ‘Psittacine’ or ‘Psittaciformes’. Citations within papers were explored as well as species-specific case studies. Overall, 188 scientific articles and books were screened, leading to the compilation of a list of 28 welfare issues (Table 1).

**Table 1.** The initial unranked list of parrot welfare issues identified through an informal literature review and presented to participants (n = 26) in the first online survey round

	Welfare Issue	References
1	Unbalanced or nutritionally deficient diet	Koutsos *et al.* (2001 ^2^), Perpiñán (2015 ^1^)
2	Lack of species-specific dietary requirements	Koutsos *et al.* (2001 ^2^), Gelis (2011 ^4^), Peron and Grosset (2013 ^2^)
3	Over-supplementation of the diet	Kalmer *et al.* (2010 ^1^), Peron and Grosset (2013 ^2^)
4	The promotion of seed and other incomplete diets as ‘whole’ diets	Ullrey *et al.* (1991 ^1^), Engebretson (2006 ^2^), Gelis (2011 ^4^), Brightsmith (2012 ^1^)
5	Unbalanced or nutritionally deficient hand feeding formulae	Cornejo *et al.* (2013 ^1^, 2021 ^1^, 2022 ^1^)
6	The hand-rearing of parrot chicks	Williams *et al.* (2017 ^1^), Grant *et al.* (2017 ^1^), Henley (2018 ^1^)
7	Limited to no foraging opportunities	Meehan *et al.* (2003b ^1^), Smith *et al.* (2021 ^1^), Mellor *et al.* (2021 ^1^), Beekmans (2023 ^1^)
8	Insufficient light exposure and/or no provision of UV lighting	Lupu and Robins (2013 ^1^), Vickery and Hollwarth (2021 ^1^), Baukhagen and Engell (2022 ^2^), Nightengale *et al.* (2022 ^2^)
9	Unsuitable cage/aviary/enclosure location	Cussen and Mench (2015 ^1^), Greenwell and Montrose (2017 ^1^), Peng and Broom (2021 ^1^)
10	Inadequate cage/aviary/enclosure size	Polverino *et al.* (2012 ^1^), Larcombe *et al.* (2015 ^1^), Peng and Broom (2021 ^1^)
11	Inadequate perches (e.g. with respect to number, size, material, variety, and/or location)	Mench *et al.* (2018 ^3^), Kaplan (2021 ^1^), Miesle (2021 ^3^)
12	Access and/or exposure to toxic foods, plants, chemicals and other harmful objects	Lightfoot and Yeager (2008 ^2^), Speer (2015 ^3^), Vickery and Hollwarth (2021 ^1^), Peng and Broom (2021 ^1^)
13	Unsanitary cage/aviary/enclosure conditions (e.g. resulting from improper cleaning and environmental [e.g. ventilation, humidity] control)	Bandyopadhyay (2017 ^3^), Greenwell and Montrose (2017 ^1^), Peng and Broom (2021 ^1^), Schwartz and Beaufrère (2022 ^3^)
14	Aspergillosis (a respiratory fungal disease caused primarily by fungal organisms of the genus Aspergillus and to a lesser extent with fungi of other genera such a Penicillium and Mucor)	Jones and Orosz (2000 ^1^), Beernaert *et al.* (2010 ^3^), Carrasco and Forbes (2016 ^1^)
15	Avian chlamydiosis (a zoonotic disease also known as psittacosis or ornithosis primarily caused by the bacterium Chlamydia)	Sachse *et al.* (2015 ^1^), Balsamo *et al.* (2017 ^2^), Ravichandran *et al.* (2021 ^2^)
16	Avian polyomavirus (APV), previously referred to as Budgerigar Fledgling Disease (BFD) (an inflammatory virus affecting juveniles in several parrot species)	Szweda *et al.* (2011 ^2^), Padzil *et al.* (2017 ^2^), Riaz *et al.* (2019 ^1^)
17	Psittacine Beak and Feather Disease (PBFD) (a viral disease belonging to the genus Circovirus, causing progressive feather, claw and beak malformation and necrosis)	Rahaus and Wolff (2003 ^1^), Rahaus *et al.* (2008 ^1^), Fogell *et al.* (2016 ^2^)
18	Proventricular Dilation Disease (PDD)/Avian Bornaviral Ganglioneuritis (ABG) caused by Avian Bornavirus (ABV) (an inflammatory disease characterized by proventricular dilation and blockage of the passage of digesta)	Tizard *et al.* (2017 ^2^), Boatright-Horowitz (2020 ^2^)
19	Parrot owner/carer unwillingness or inability to seek and/or implement veterinary and/or behavioural advice	Engebretson (2006 ^2^), Gaskins and Bergman (2011^1^), Baukhagen and Engell (2022 ^2^)
20	Abnormal behaviours (ABs), abnormal repetitive behaviours (ARBs) and stereotypic behaviour	Fox (2006 ^3^), Schmid *et al.* (2006 ^1^), Garner *et al.* (2006 ^1^), Cussen and Mench (2015 ^1^)
21	Absence of predictability, routine and control	Henley (2018 ^1^), Baukhagen and Engell (2022 ^2^)
22	Insufficient and/or inadequate environmental enrichment	Acharya and Rault (2020 ^1^), Stevens *et al.* (2021 ^1^)
23	Social isolation from conspecifics (i.e. other parrots)	Kalmer *et al.* (2010 ^1^), Aydinonat *et al.* (2014 ^1^), Williams *et al.* (2017 ^1^)
24	Overbonding with owner/carer(s)	Van Sant (2006 ^3^), Scagnelli and Tully (2017 ^1^)
25	Incompatible social groups	Andrews (2022 ^3^)
26	Wing clipping	Kubiak (2015 ^4^), Larcombe *et al.* (2015 ^1^)
27	Limited ability or inability to carry out feather maintenance	Bergman and Reinisch (2006 ^3^), Rubinstein and Lightfoot (2012 ^1^), Van Zeeland and Schoemaker (2014 ^2^)
28	Rehoming of parrot(s)	Acharya and Rault (2020 ^1^), Baukhagen and Engell (2022 ^2^)

1 Indicates peer-reviewed journal publications;

2 specifies if these were literature reviews;

3 indicates books or book chapters;

4 denotes conference proceedings, doctoral theses, or veterinary magazine publications.

#### Priority identification surveys

Two priority identification surveys (Surveys 1 and 2) were hosted on the JISC Online Survey platform from May–July 2023 (see Supplementary material). Survey questions were designed by the research team and piloted prior to study commencement to refine question structure and clarity.

Survey 1 collected participant demographic information (age, gender, occupation, highest level of education and years of experience with captive parrots) and presented participants with the 28 welfare issues identified from the informal literature review (Table 1). Participants were asked to rank the 28 welfare issues according to three criteria, adopted from Rioja-Lang *et al.* (2019a,b), as follows:

- *Severity* –the severity in which the welfare concern is likely to or commonly presents in the participant’s opinion;
- *Duration* –the duration in which the welfare concern is likely to or commonly affects the individual in the participant’s opinion;
- *Prevalence* –the perceived proportion of affected individuals in the participant’s opinion.

Issues were ranked on a six-point scale as follows: 1 = mild to 6 = debilitating (severity); 1 = fleeting to 6 = the entire duration of the individual’s life (duration); and 1 = rare to 6 = universally present in the population (prevalence). Rank questions were mandatory, with optional open-ended response questions after each issue to allow participants to provide commentary on their ranking if desired. At the end of Survey 1, participants were provided with the opportunity to add any new welfare issues, rank according to the criteria and share commentary. Out of the 93 individuals contacted, 26 participants completed Survey 1, representing a response rate of 28%.

Upon preliminary analysis of Survey 1, several participants provided valuable additional feedback regarding the initial list of welfare issues. As a result, a sub-survey (Survey 1B) was developed to include eight additional welfare issues (Table 2) and four re-described welfare issues for participants to rank according to the criteria and provide commentary if they so desired. Survey 1B was sent to all who had completed survey 1 and was completed by 12 participants.

**Table 2.** New captive parrot welfare issues added by participants in issue prioritisation survey 1 (n = 26) and included in survey 1B (n = 12)

	Welfare issue
1.	Enforced prolonged periods of complete darkness as a result of cages being covered and/or kept in rooms with no light regulation/ no transitionary period
2.	Inadequate housing temperature
3.	Inappropriate and/or unsafe toys (i.e. improper materials, potential physical harm from damaged toys)
4.	Improper breeding management, e.g. genetic testing, disease testing, husbandry management
5.	Inability to carry out mating behaviours
6.	Insufficient legislation and/or regulation surrounding the sale of parrots in commercial settings e.g. pet stores, auction houses and shows resulting in unregulated welfare practices
7.	Non-avian vets treating avian (parrot) species, often resulting in misdiagnosis or improper care
8.	Lack of shelters or rescues with the ability, capacity or knowledge to take in and re-home birds to suitable homes for the duration of the birds’ lives

Participants’ rankings of each issue from Surveys 1 and 1B were downloaded to Microsoft Excel® and the mean rank score in terms of severity, duration, and prevalence was calculated for each welfare issue. All welfare issues with a mean response score of 4 or more were included in the second survey round (Survey 2). These are listed from highest ranking issue to lowest in Table 3.

**Table 3.** Mean (± SD) scores and rank order for parrot welfare issues scoring at least 4.0 or above for severity, duration and prevalence after survey rounds 1 (n = 26) and 1B (n = 12)

Rank Order	Severity	Mean Score (± SD)	Duration	Mean Score (± SD)	Prevalence	Mean Score (±SD)
1	Lack of environmental enrichment resulting in a cognitively unchallenging environment	5.17 (± 0.83)	Inadequate cage/aviary/enclosure size (restricting movement, flight ability, foraging and other natural behaviours)	5.67 (± 0.49)	Inadequate cage/aviary/enclosure size (restricting movement, flight ability, foraging and other natural behaviours)	5.08 (± 1.08)
2	Insufficient legislation and/or regulation surrounding the sale of parrots in commercial settings e.g. pet stores, auction houses and shows resulting in unregulated welfare practices.	5.08 (± 0.9)	Unsuitable cage/aviary/enclosure location (resulting in sleep deprivation, hypervigilance, physical harm)	5.5 (± 0.5)	Insufficient legislation and/or regulation surrounding the sale of parrots in commercial settings e.g. pet stores, auction houses and shows resulting in unregulated welfare practices.	4.83 (± 1.59)
3	Inadequate cage/aviary/enclosure size (restricting movement, flight ability, foraging and other natural behaviours)	5.08 (± 0.9)	Lack of environmental enrichment resulting in a cognitively unchallenging environment	5.5 (± 0.9)	Lack of environmental enrichment resulting in a cognitively unchallenging environment	4.75 (± 0.97)
4	Psittacine Beak and Feather Disease (PBFD) (a viral disease belonging to the genus Circovirus, causing progressive feather, claw and beak malformation and necrosis)	4.78 (± 1.78)	Social isolation from conspecifics (i.e. other parrots)	5.35 (± 0.98)	Inability to carry out mating behaviours	4.67 (± 1.5)
5	Social isolation from conspecifics (i.e. other parrots)	4.77 (± 1.27)	The hand-rearing of parrot chicks (to include the selling of un-weaned birds to inexperienced owners/handlers for the purpose of hand-rearing)	5.17 (± 1.11)	Social isolation from conspecifics (i.e. other parrots)	4.65 (± 1.29)
6	Proventricular Dilation Disease (PDD)/Avian Bornaviral Ganglioneuritis (ABG) caused by Avian Bornavirus (ABV) (an inflammatory disease characterized by proventricular dilation and blockage of the passage of digesta)	4.73 (± 1.39)	Improper breeding management, e.g. genetic testing, disease testing, husbandry management	5.17 (± 0.83)	Limited to no foraging opportunities	4.56 (± 1.12)
7	Parrot owner/carer unwillingness or inability to seek and/or implement veterinary and/or behavioural advice	4.62 (± 1.1)	Unbalanced or nutritionally deficient diet	5.12 (± 1.14)	Lack of species-specific dietary requirements	4.54 (± 1.21)
8	Abnormal behaviours (ABs), abnormal repetitive behaviours (ARBs) and stereotypic behaviour	4.58 (± 1.03)	Lack of species-specific dietary requirements	5.04 (± 1.18)	Unbalanced or nutritionally deficient diet	4.5 (± 1.1)
9	The hand-rearing of parrot chicks (to include the selling of un-weaned birds to inexperienced owners/handlers for the purpose of hand-rearing)	4.5 (± 1.38)	Limited to no foraging opportunities	5 (± 1.12)	Insufficient light exposure and/or no provision of UV lighting	4.44 (± 1.26)
10	Unsuitable cage/aviary/enclosure location (resulting in sleep deprivation, hypervigilance, physical harm)	4.5 (± 1.37)	Insufficient light exposure and/or no provision of UV lighting	5 (± 1.38)	Unsuitable cage/aviary/enclosure location (resulting in sleep deprivation, hypervigilance, physical harm)	4.33 (± 1.33)
11	Wing clipping resulting in physical harm and restricted flight	4.5 (± 1.38)	Abnormal behaviours (ABs), abnormal repetitive behaviours (ARBs) and stereotypic behaviour	5 (± 0.98)	The promotion of seed and other incomplete diets as ‘whole’ diets	4.21 (± 1.38)
12	Avian polyomavirus (APV), previously referred to as Budgerigar Fledgling Disease (BFD) (an inflammatory virus affecting juveniles in several parrot species)	4.45 (± 1.74)	Overbonding with owner/carer(s)	5 (± 1.02)	Improper breeding management, e.g. genetic testing, disease testing, husbandry management	4.17 (± 1.75)
13	Aspergillosis (a respiratory fungal disease caused primarily by fungal organisms of the genus Aspergillus and to a lesser extent with fungi of other genera such a Penicillium and Mucor)	4.44 (± 1.76)	Parrot owner/carer unwillingness or inability to seek and/or implement veterinary and/or behavioural advice	4.96 (± 1.37)	Lack of shelters or rescues with the ability, capacity or knowledge to take in and re-home birds to suitable homes for the duration of the birds’ lives	4.17 (± 1.4)
14	Non-avian vets treating avian (parrot) species, often resulting in misdiagnosis or improper care	4.25 (± 1.66)	Insufficient legislation and/or regulation surrounding the sale of parrots in commercial settings e.g. pet stores, auction houses and shows resulting in unregulated welfare practices.	4.92 (± 1.68)	Abnormal behaviours (ABs), abnormal repetitive behaviours (ARBs) and stereotypic behaviour	4.12 (± 1.51)
15	Limited to no foraging opportunities	4.24 (± 1.16)	Psittacine Beak and Feather Disease (PBFD) (a viral disease belonging to the genus Circovirus, causing progressive feather, claw and beak malformation and necrosis)	4.91 (± 1.38)	Parrot owner/carer unwillingness or inability to seek and/or implement veterinary and/or behavioural advice	4.04 (± 1.48)
16	Access and/or exposure to toxic foods, plants, chemicals and other harmful objects	4.24 (± 1.94)	The promotion of seed and other incomplete diets as ‘whole’ diets	4.79 (± 1.5)	Non-avian vets treating avian (parrot) species, often resulting in misdiagnosis or improper care	4 (± 1.13)
17	Unbalanced or nutritionally deficient diet	4.19 (± 1.13)	Inadequate perches (e.g. with respect to number, size, material, variety, and/or location)	4.77 (± 1.45)		
18	Overbonding with owner/carer(s)	4.19 (± 1.1)	Unsanitary cage/aviary/enclosure conditions (e.g. resulting from improper cleaning and environmental [e.g. ventilation, humidity] control)	4.69 (± 1.44)		
19	The promotion of seed and other incomplete diets as ‘whole’ diets	4.17 (± 1.34)	Proventricular Dilation Disease (PDD)/Avian Bornaviral Ganglioneuritis (ABG) caused by Avian Bornavirus (ABV) (an inflammatory disease characterised by proventricular dilation and blockage of the passage of digesta)	4.61 (± 1.47)		
20	Insufficient light exposure and/or no provision of UV lighting	4.16 (± 1.4)	Wing clipping resulting in physical harm and restricted flight	4.58 (± 1.08)		
21	Improper breeding management, e.g. genetic testing, disease testing, husbandry management	4.16 (± 1.56)	Lack of shelters or rescues with the ability, capacity or knowledge to take in and re-home birds to suitable homes for the duration of the birds’ lives	4.58 (± 1.51)		
22	Lack of species-specific dietary requirements	4.08 (± 1.35)	Inability to carry out mating behaviours	4.42 (± 1.56)		
23	Inadequate housing temperature	4 (± 1.35)	Non-avian vets treating avian (parrot) species, often resulting in misdiagnosis or improper care	4.42 (± 1.44)		
24	Lack of shelters or rescues with the ability, capacity or knowledge to take in and re-home birds to suitable homes for the duration of the birds’ lives	4 (± 1.41)	Absence of predictability, routine and control	4.32 (± 1.44)		
25			Inadequate housing temperature	4.08 (± 1.44)		
26			Incompatible social groups	4.04 (± 1.72)		
27			Enforced prolonged periods of complete darkness as a result of cages being covered and/or kept in rooms with no light regulation/ no transitionary period.	4 (± 1.3)		

Survey 2 was sent to those who had completed Survey 1 and was completed by 14 participants. Participants were presented with the list of welfare issues carried forward within each category (severity, duration and prevalence) and asked whether they:

- agreed with the ranking of the welfare issue;
- disagreed with the ranking of the welfare issue as it should be higher or;
- disagreed with the ranking of the welfare issue as it should be lower.

#### Analysis of survey responses

Participant agreement on the ranking placements for the welfare issues in Survey 2 was analysed using Fleiss Kappa in R Studio (R Studio Team 2023).

Open responses provided by participants in Surveys 1 and 2 were processed with deductive content analysis to identify recurrent themes raised by participants in justifying their rankings (Elo & Kyngäs 2008). This analysis was conducted to obtain the literal meaning of participants’ responses, rather than creating interpretations or inferring wider meanings. Within this process, similarities in responses were grouped and described under a set of themes. Recurrence of theme use across participant responses was noted to generate a count of prevalence of themes referenced across participants. Minor spelling errors within comments were edited for presentation.

#### Online workshops

The final phase of the study consisted of two 2-h online workshops conducted over Microsoft Teams® in November 2023. Those who completed Survey 2 (n = 14) were invited to participate. Of these, seven participants attended the workshops (n = 3 in one, n = 4 in the other; two workshops were held to accommodate participant availability across time zones). Workshops were facilitated by RC and BV. Discussions focused first on the welfare issues in terms of their severity, duration and prevalence. During the workshops, participants discussed: whether they concurred with the rank order from previous stages of the study; whether issues should be re-ordered; and whether any issues had been overlooked. Once participants agreed on the final rankings for severity, duration, and prevalence, they worked to generate a top 10 highest priority list overall. Consensus was deemed to have been achieved during the workshop discussions when each participant verbally confirmed agreement on the issues and their respective rank orders for each of the final four lists of severity, duration, prevalence, and top 10 overall.

As the workshop was conducted twice with two sub-groups of participants, the agreed rankings from the first workshop were shared to participants in the second workshop via email prior to commencement of the second workshop. Participants in the second workshop were encouraged to take these results into account during their discussions. Again, consensus was considered to have been reached when each participant agreed on the rank order for each category. Final agreed lists from workshop 2 were then re-shared via email with participants from workshop 1, who were asked to indicate their agreement or disagreement with the final lists. This approach allowed us to assess the level of consensus reached among all seven workshop participants.

## Results

### Demographics of starting participant pool

We present broad descriptions of the demographic characteristics of the participants who shared their views in Survey 1 but do not provide a detailed breakdown of demographics at subsequent phases to protect participant anonymity, though we note that expert roles remained varied in later study phases. Twenty-six experts and sector professionals completed Survey 1; these were predominantly veterinarians (46%), zoo affiliates (19%) or academics (15%), followed by behaviourists (8%), animal welfare public affairs and publishing personnel (8%), and those holding non-animal roles at the time of the study but who had previous and recent extensive parrot experience (4%). Most participants had professional qualifications (34%; e.g. DVM, MD, DO, JD) or postgraduate degrees (31%; Master’s or PhD); seven (27%) had a bachelor’s degree and two (8%) participants held other qualifications. Most had between 3–10 (31%) or 11–20 (27%) years of experience working with parrots; 19% had worked with parrots for 21–30 years and nearly a quarter (23%) had over 30 years of work experience. Participants were predominantly female (65%), between the ages of 25–34 and 35–44 (23% each), and resided in the UK (46%) or the USA (34%). By the workshops round, four participants were female and three were veterinarians.

### Surveys 1 and 1B

Of the 36 welfare issues (28 from the initial list plus the additional eight contributed by participants in Survey 1; see Tables 1 and 2), 24 issues scored a mean response score of 4 or more for severity, 27 for duration, and 16 for prevalence; these were carried forward into Survey 2 (Table 3).

Of the original participant pool, 18 participants provided commentary to justify their rankings, with a total of 192 comments left by participants across Surveys 1 and 1B. These comments touched upon four key topics in their focus: ‘behaviour’ (31% of comments referencing behaviour-related issues), ‘health’ (23%), ‘nutrition’ (22%), and ‘environment’ (18%), with the remaining 5% comments raising ‘miscellaneous’ issues not easily classified into the previous four). Within discussion of these topics, participants noted that they either: ‘unconditionally agreed’ that the issue had been ranked appropriately in terms of severity, duration, or prevalence; ‘circumstantially agreed’ (i.e. agreed about the issue but this was contingent on context or external factors), ‘disagreed’ that the issue was of importance; or were ‘unclear’ about the issue. Overall, participant commentary most often unconditionally or circumstantially agreed with the welfare issues presented (see Table 4 for themes and exemplar participant comments).

**Table 4.** Themes, description, and exemplar quotations from participants commenting on parrot welfare issues in priority issue ranking surveys 1 (n = 26) and 1B (n = 12)

Themes	Description	Example commentary
**Unconditional agreement**	Comments of unwavering support or endorsement for the welfare issue without any conditions or reservations. Often expands on why the issue is seen to be a problem.	“Almost every bird displayed stereotypy ranging from feather pulling (this was surprisingly rare and isolated to two birds), head swinging, repetitive actions with no purpose. One bird in particular would lick a certain spot of metal for hours on end.” “Very few caregivers feed a species appropriate food” *“*A serious welfare issue” “I think this practice is one of the most inappropriate and appalling things aviculture allows”
**Circumstantial agreement**	Comments reflecting agreement contingent upon specific conditions, circumstances, or affecting a proportion of the population.	“The welfare implication here depends on whether the rehoming results in improved care and housing as a result and how frequently it happens. the welfare impact can be highly variable, but is common especially for longer lived species.” “It is probably more common in smaller parrots (budgies, lovebirds etc) who are typically fed an all seed diet” “Depends on the respective country’s regulations”
**Disagreement**	Comments expressing disagreement that the welfare issue is serious, or offering differing opinions, or conflicting viewpoints. Often noting that the issue was more of a problem in the past.	“A single pet parrot can easily come to identify with its steward(s) as conspecifics - social interaction does not require communication/interaction with the same species in this light.” “Not seen this since the changes in the way birds are bought and sold.” “I think this has reduced a lot now[.] Individual isolation hand rearing is less frequent” “Rarely see it much any more or at least in my demographic area”
**Unclear**	Comments that are ambiguous, lacking clarity, or open to interpretation, presenting challenges in discerning which theme they fall under	“Very hard to define this, or understand it is happening… or the consequences.” “Unusual!”

### Survey 2

Fleiss kappa results indicated poor agreement for ranking of the welfare issues across the lists in Survey 2 (*K* = 0.014 for severity, *K* = 0.017 for duration and *K* = 0.008 for prevalence) and this was also represented in feedback on ranks of specific issues. For example, regarding the severity ranking (7th) of ‘Parrot owner/carer unwillingness or inability to seek and/or implement veterinary and/or behavioural advice’, opinions were divided among agreement (57%), disagreement that the ranking should be lower (29%), and disagreement that it should be higher (14%). Opinions on duration rankings were similarly divided; for example, participants were split nearly evenly between agreement (43%) with the 6th ranked duration position of ‘improper breeding management, e.g. genetic testing, disease testing, husbandry management’ and disagreement that the ranking should be lower (50%; with 7.1% of participants suggesting that it should be higher). Opinions on prevalence were likewise often divided; for example, participant opinion was split on the prevalence ranking (11th) of ‘the promotion of seed and other incomplete whole diets’, with 36% agreeing with the current ranking, 21% disagreeing and indicating that the ranking should be lower, and 43% disagreeing and indicating that the ranking should be higher.

### Online workshops

During the workshop discussions, participants assessed the ranked lists generated from Survey 2 to conclude their final lists and ranking of issues, modifying the order of the lists and the issues within them where they felt this was warranted. Participants also re-categorised welfare issues already present in the list under wider ‘umbrella’ terms (e.g. Nutrition – to include all nutritional related welfare issues) to encompass a wider variety of issues participants believed were interlinked. New welfare issues were also introduced at this stage (e.g. geriatric care, pain recognition). Re-categorisation of welfare issues was conducted with relative ease, with only minor disagreement among participants that was resolved during the workshops. For example, participants debated whether ‘Environmental ability to express behaviours’, ‘Development of normal behaviour’ and ‘Abnormal behaviours (ABs), abnormal repetitive behaviours (ARBs) and stereotypic behaviour’ should be re-categorised under one welfare issue, but after some discussion participants agreed that although certain aspects of these welfare issues were causational to one another, they were in themselves each notable welfare issues which may develop independently.

The final priority lists constructed by participants in the online workshops are presented in Table 5. Across the priority lists, welfare issues such as ‘environmental ability to express behaviours’, ‘nutrition’, ‘social isolation from conspecifics’, and ‘housing’ routinely ranked among the top three welfare issues. Consensus on the order of the top three welfare issues was reached without disagreement among participants. Among the remaining ranks, disagreement among participants was uncommon, with minimal dissention throughout the online workshops to establish rank order. By conclusion of the study, six of the seven participants verified their final agreement with all lists, resulting in 86% consensus amongst the experts and sector professionals. The remaining participant (from workshop 1) did not respond to the follow-up email to confirm or deny their agreement.

**Table 5.** Final captive parrot welfare issue rankings (in terms of severity, duration, prevalence, and overall) after an iterative modified Delphi expert consultation study (n = 26 experts at study start, 7 experts by study conclusion)

Rank Order	Severity	Duration	Prevalence	Top 10 Overall
1	Environmental ability to express behaviours (e.g. provision of environmental enrichment, flight)	Development of normal behaviour (e.g. parental deprivation/hand-rearing of chicks, sourcing/acquisition; causes downstream effects throughout life)	Nutrition (i.e. how and what birds are fed, e.g. foraging, lack of species-specific diets, obesity)	Lack of (prospective and current) owner knowledge, education, and access to proper veterinary/behaviour support
2	Nutrition (i.e. how and what birds are fed, e.g. foraging, lack of species-specific diets, obesity)	Social isolation from conspecifics	Environmental ability to express behaviours (e.g. provision of environmental enrichment, flight)	Social isolation throughout life
3	Social isolation from conspecifics	Nutrition (i.e. how and what birds are fed, e.g.foraging, lack of species-specific diets, obesity)	Housing (e.g. Inadequate cage/aviary/enclosure size + unsuitable cage/aviary/enclosure location + appropriate temperature)	Housing (e.g. Inadequate cage/aviary/enclosure size + unsuitable cage/aviary/enclosure location + appropriate temperature + lighting)
4	Housing (e.g. Inadequate cage/aviary/enclosure size + unsuitable cage/aviary/enclosure location + appropriate temperature)	Housing (e.g. Inadequate cage/aviary/enclosure size + unsuitable cage/aviary/enclosure location + appropriate temperature)	Development of normal behaviour (e.g. parental deprivation/hand-rearing of chicks, sourcing/acquisition; causes downstream effects throughout life)	Environmental ability to express behaviours (e.g. provision of environmental enrichment, flight)
5	Lack of owner knowledge, education, and access to proper vet and/or behaviour support (e.g. hand rearing)	Environmental ability to express behaviours (e.g. provision of environmental enrichment, flight)	Lack of owner knowledge, education, and access to proper vet/behaviour support (e.g. hand rearing)	Nutrition (i.e. how and what birds are fed, e.g. foraging, lack of species-specific diets, obesity)
6	Development of normal behaviour (e.g. parental deprivation/hand-rearing of chicks, sourcing/acquisition; causes downstream effects throughout life)	Absence of predictability, routine and control	Social isolation from conspecifics + inability to form appropriate pair bonds	Development of normal behaviour (e.g. parental deprivation/hand-rearing of chicks, sourcing/acquisition; causes downstream effects throughout life)
7	Abnormal behaviours (ABs), abnormal repetitive behaviours (ARBs) and stereotypic behaviour	Lack of owner knowledge, education, and access to proper vet and/or behaviour support (e.g. hand rearing)	Insufficient application/enforcement of legislation and/or regulation	Lack of a ‘life plan’ for birds (e.g. end of life planning impacting decisions for euthanasia or rehoming + availability of qualified parrot rescues, dealing with unwanted birds, e.g. little birds released)
8	Lack of veterinarian education (e.g. we don’t educate non-avian vets to treat avian species, lack of exotic-specialised vets)	Inappropriate light exposure (including overexposure or too long days/ inadequate night/day cycle)	Abnormal behaviours (ABs), abnormal repetitive behaviours (ARBs) and stereotypic behaviour	Abnormal behaviours (ABs), abnormal repetitive behaviours (ARBs) and stereotypic behaviour
9	Insufficient application/enforcement of legislation and/or regulation	Abnormal behaviours (ABs) abnormal repetitive behaviours (ARBs) and stereotypic behaviour	Lack of a ‘life plan’ for birds (e.g. end of life planning impacting decisions for euthanasia or rehoming + availability of qualified parrot rescues; dealing with unwanted birds, e.g. little birds released)	Lack of education to the veterinary profession
10	Disease, e.g. Psittacine Beak and Feather Disease (PBFD) + Proventricular Dilation Disease (PDD)/Avian Bornaviral Ganglioneuritis (ABG)	Geriatric care	Geriatric care	Insufficient application/enforcement of legislation and/or regulation

## Discussion

This study sought to determine priority welfare issues among captive parrots using a modified Delphi approach. Twenty-six participants with parrot expertise were initially recruited, and through successive rounds, four lists were generated to establish the ten highest priority welfare issues facing parrots in captivity according to severity, duration, prevalence and overall. Participants highlighted some over-arching issues as well as more specific welfare considerations, such as the role of legislation as a contributing factor to poor welfare, suggesting a need for increased enforcement and regulation of pre-existing welfare laws rather than the formulation of new laws. Similarly, a lack of education among owners and veterinary professionals was seen to contribute to welfare issues in practice (e.g. nutrition, housing, social isolation). Across all rank categories, participants focused on nutrition (e.g. inadequate diet, poor feeding practices and lack of foraging opportunities), housing (e.g. inadequate perches, temperature, light availability and size and location of cage/aviary/enclosure), environmental ability to express behaviours, development of normal behaviour and social isolation from conspecifics. In this discussion, we focus on the issues that were included in the top 10 overall priority list, based on consensus from the online workshops.

### 1. Lack of (prospective and current) owner knowledge, education, and access to proper veterinary and/or behaviour support

Participants identified lack of owner knowledge and education as the leading parrot welfare issue overall. In this respect, the expert pool in this study aligns with Baukhagen and Engell’s (2022) recommendation that “*no one should be able to purchase a parrot of any size without demonstrating they have conducted research into the care, lifespan, and personality of said parrot.*” Similar expert consultations have also highlighted lack of owner knowledge as a key precipitating factor affecting the welfare of other companion species (Rioja-Lang *et al.* 2020) Others have also observed that prospective owners often lack awareness of the realities of parrot ownership, an issue amplified by the proliferation of inaccurate information (Hoppes & Gray 2010; Grant *et al.* 2017). It is likely that this issue is one of several underlying, systemic factors affecting the welfare of companion parrots.

Parrots can exhibit behaviours which owners may find challenging, including vocalisations such as those described as ‘screaming’ and the display of apparently abnormal behaviours and stereotypies. Behaviours considered undesirable include aggression towards other members of the household such as attacking the partners of their caretakers, defaecation around the house, and the destruction of property; such behaviours can often lead to the bird being relinquished (Anderson 2003; Henley 2018). Efforts to address these challenges could involve targeted education by leading charities, veterinary practices and pet stores for prospective owners, enabling them to make informed decisions regarding the choice of species that best suits their lifestyles, or reconsidering the choice of a parrot altogether. Increasing awareness of parrot welfare issues may also reduce relinquishment of parrots, decreasing the number of parrots within rescue centres (Engebretson 2006; Baukhagen & Engell 2022).

In the UK, there is a notable scarcity of avian veterinarians, with approximately 104 identified avian veterinarians reported by The Parrot Society (2023) in comparison to the estimated 1.6 million ornamental birds in the UK (FEDIAF 2023), potentially resulting in owners having to register with and travel farther to specialist veterinary practices. Goins and Hanlon (2021) found that 34% of exotic pet owners never sought veterinary care due to a lack of local veterinary services, even though four out of five veterinary professionals in small or mixed animal practices advised they were willing to treat exotic pets. Goins and Hanlon (2021) also suggested perceived lack of species-specific competence as a significant factor in failure to engage with appropriate veterinary services. This is supported by García-San Román *et al.* (2023), who suggested pet owners prefer veterinarians who specialise and have post-graduate training in their pet’s health issues.

### 2. Social isolation throughout life

Parrots are highly social species, largely living in flocks (except for solitary species such as the kākāpo, *Strigops habroptilus*), yet they are commonly housed alone in captive settings (Engebretson 2006; Meehan & Mench 2006). Individual housing contributes to adverse consequences for parrot behaviour and welfare: solo-housed parrots demonstrate increased stereotypic behaviour and less preening (Williams *et al.* 2017) and show increased biting and food stealing (Tygesen & Forkman 2023). Others have shown social isolation to be a contributing factor in feather-damaging behaviour in young and orange-winged Amazon parrots (*Amazona Amazonica)* (Meehan *et al.* 2003a; Garner *et al.* 2006). Social isolation has also been linked to shortened telomere length (associated with aging) in African grey parrots (*Psittacus Erithacus*), with the telomere length of single-housed birds at nine years of age being comparable to pair-housed birds 23 years older than themselves (Aydinonat *et al.* 2014).

### 3. Housing (e.g. Inadequate cage/aviary/enclosure size + unsuitable cage/aviary/enclosure location + appropriate temperature + lighting)

Participants in this study raised concerns regarding the living conditions of parrots, involving a restricted environment, improper cage location, ultraviolet light exposure and a lack of environmental control. In the UK, there is limited guidance for parrot housing requirements, except that associated with birds used for breeding and sale, which are covered by The Animal Welfare (Licensing of Activities Involving Animals) (England) Regulations (2018) (DEFRA 2018). This guidance specifies “*For birds housed singly that spend the majority of their time in a cage, the cage width must be a minimum of twice flying wingspan, and the depth and height a minimum of one and a half times the birds flying wingspan. A pair of birds must have enough space to fly past each other with the depth being increased to a minimum of 2x flying wingspan.”* Further, species-by-species requirements are covered by the statutory guidance for local authorities (DEFRA 2024). However, with respect to parrots in other circumstances, owners are simply expected to provide ‘a suitable environment’ according to the Animal Welfare Act (2006). In this study, participants maintained clear concerns that unsuitable, restrictive environments posed serious threats to parrot welfare. A restrictive environment can profoundly impact a parrot’s physical and mental well-being, hindering natural behaviours and locomotor activities, such as foraging, flight, grooming and play (Engebretson 2006; van Zeeland *et al.* 2009). Behavioural restriction may manifest in the development of repetitive oral behaviours (e.g. feather picking), locomotor stereotypies (e.g. route tracing) and inter- and intra-specific aggression (Meehan *et al.* 2004; Meehan & Mench 2006), as well as physical disorders associated with a sedentary lifestyle such as atherosclerosis and obesity (Beaufrère 2013; Chitty 2023; Burns 2024).

Cage location was also discussed as a threat to welfare; proximity to potential predators such as cats and dogs and other aversive stimuli have been linked to stress-induced abnormal behaviours such as feather picking or plucking and to excessive vocalisation (Bergman & Reinisch 2006; Garner *et al.* 2006; Jayson *et al.* 2014). Improper cage placement can also trigger expression of fear, including fear-based aggression and hypervigilance, leading to undesirable behaviours such as biting and ‘screaming’, escape attempts, and injury due to the bird’s inability to remove itself from potential danger (Wilson & Luescher 2006).

Participants also discussed concerns regarding inadequate exposure to ultraviolet (UV) light. There appears to be limited understanding of UV requirements among avian species, including parrots (Stanford 2006; Ross *et al.* 2013), despite its role in maintaining feather quality, calcium metabolism, vitamin D synthesis and colour perception (Stanford 2006; Berg & Bennett 2016; Baukhagen & Engell 2022). Further research is needed to determine UV requirements, for example, the optimal duration of UV exposure for captive parrots and distance from the light source for effective exposure. Baukhagen and Engell (2022) suggest a potential link between vitamin D deficiency and mood, proposing that deficiency may lead to a depressed mood, as observed in humans (Parker *et al.* 2017).

Participants also raised concerns regarding lack of control and predictability in the captive environment, highlighting the disparity between decision-making demands in the wild which engage a parrot’s mind and require significant mental effort and the potentially monotonous and predictable conditions associated with captivity (Mellor *et al.* 2021). This discrepancy raises concerns for cognitive functioning: for example, Baukhagen and Engell (2022) discussed the possibility that eliminating freedom to make choices may diminish a captive parrot’s cognitive abilities and change their neuroanatomy, as observed in other species such as captive songbirds (Tarr *et al.* 2009).

### 4. Environmental ability to express behaviours (e.g. provision of environmental enrichment, flight)

Captive environments limit opportunities to express behaviours that form the free-living parrot’s behavioural repertoire (Kalmer 2011). For example, wild Puerto Rican Amazons (*Amazona vittata*) spend four to six hours per day foraging and are known to ingest the fruit, leaves, bark, vines, and/or other portions of at least 58 species of indigenous plants (Meehan & Mench 2006). However, captive environments are often predictable in resource distribution and poor in physical and mental stimulation, leading to a reduction or prevention of natural behaviours (Miglioli & da Silva Vasconcellos 2021).

Environmental enrichment aims to increase opportunity to express these natural patterns of behaviour and, in turn, may also reduce abnormal behaviours and stereotypies, discourage inactivity, provide mental stimulation, and decrease fear responses (Mason *et al.* 2007; Rodríguez-López 2016). However, further study into variations of enrichment, such as flight and social play (Diamond *et al.* 2006; Rodríguez-López 2016), is warranted. Flight behaviour is often denied to varying degrees in captivity, and whilst enrichment to increase locomotion has been widely discussed (Meehan *et al.* 2004; Clyvia *et al.* 2015; Assis *et al.* 2016), specific flight enrichment techniques (e.g. free-flying) have yet to be explored fully in captive parrots, with current studies focusing on the use of free-flight in pre-release training activities (Woodman *et al.* 2021; Franzone *et al.* 2022).

### 5. Nutrition (i.e. how and what birds are fed, e.g. foraging, lack of species-specific diets, obesity)

Nutrition is one of the most challenging aspects of captive parrot care (Peron & Grosset 2013; Baukhagen & Engell 2022). The requirements for nutritional balance as well as trace nutrients have yet to be obtained for many species (see Kalmer *et al.* 2010; Peron & Grosset 2013) and poultry requirements remain the default when establishing captive diets (Koutsos *et al.* 2001; Peron & Grosset 2013). Whilst the importance of an ‘ancestral diet’ (i.e. flowers, fruits, nuts, seeds, grasses, insects and other plant material) for captive parrots has received some consideration, commercial feeds neither reflect this nor current understanding of parrot nutrition (Koutsos *et al.* 2001; Baukhagen & Engell 2022). For instance, commercial seed mixes remain commonplace and are often misleadingly marketed as ‘complete’ diets suitable across species, leaving specialist feeders such as nectivorous species, including lories (*loriinae)* and lorikeets (*Trichoglossus* moluccanus), particularly at risk (Ullrey *et al.* 1991; Gelis 2011; Brightsmith 2012).

The consequences of feeding poorly designed seed mixes to parrots can be severe, due primarily to their high levels of fat and protein and lack of vitamins A, D, K and E, calcium, and essential amino acids such as lysine and methionine (Harrison & McDonald 2006; Hess 2020). Inappropriate diet formulation can result in disorders such as obesity, atherosclerosis, fatty liver disease, and deficiencies in key vitamins and minerals required for normal bodily function (see Hess 2020). For example, chronically low levels of vitamin A (hypovitaminosis A) can result in respiratory tract disease (Zwart & Samour 2021), feather picking, skin problems, and overall decline in feather growth and quality (van Zeeland *et al.* 2009; Peron & Grosset 2013; Samour 2015).

Our participants also discussed nutrition as a welfare issue in relation to how parrots receive their food. Foraging – which includes the search for food, selection, procurement, manipulation, and consumption – is often restricted in captivity, with captive diets contributing to reduced feeding times (van Zeeland *et al.* 2013, 2023). For example, whilst pellets may be a better nutritional alternative to seed diets (as they provide a more balanced diet than may be achieved by selecting from a seed mix), they offer little variety in texture, colour and flavour, significantly reducing overall feed time (van Zeeland *et al.* 2023). Wild parrots typically spend between 4 and 8 h per day on foraging, whereas parrots in captivity often spend less than 1 h per day foraging (Baukhagen & Engell 2022; Beekmans *et al.* 2023).

Parrots have been found to work for food even when identical food is freely available (for example, contra-freeloading, see van Zeeland *et al.* 2023), suggesting they value the behaviours involved in selecting and acquiring food. The inability to carry out species-specific feeding behaviours may result in the expression of abnormal behaviours and stereotypies, including oral stereotypies, such as wire chewing, tongue playing, food manipulation or dribbling, and feather-damaging behaviours (Meehan & Mench 2006; van Zeeland *et al.* 2013). Foraging enrichment can reduce the occurrence of these behaviours as it stimulates exploration and can significantly increase time spent foraging up to 2–3 h a day (Meehan *et al.* 2003b; Beekmans *et al.* 2023).

### 6. Development of normal behaviour (e.g. parental deprivation/hand-rearing of chicks, sourcing/acquisition; causing downstream effects throughout life)

Wild parrots would normally develop patterns of behaviour in a social environment, initially made up of their parents and siblings, but in time with the wider social group. This allows development of important behaviours, such as species and sexual recognition, feeding preferences, and behavioural skills to respond to social challenges. Parent rearing in captivity can mimic aspects of this early environment, but hand-rearing is commonly practiced in young, captive parrots. Hand-rearing involves separating the parrot chick from its parents (typically having been artificially incubated) and deprives the young bird of contact which allows for normal social and sexual development. As a result, hand-reared birds often show a preference for contact with humans, imprinting socially and sexually (Fox 2006). Handling neonatal parrots can also compromise their ability to respond to stress (Collette *et al.* 2000). Baukhagen and Engell (2022) suggest premature weaning has the potential to elicit lifelong negative behaviours such as increased anxiety and aggression; considering parrots’ longevity, this poses a significant welfare concern. Upon reaching adulthood, hand-reared parrots show inappropriate reproductive behaviours and abnormal sexual behaviours (such as masturbation and regurgitation onto objects or the caregiver), hypersexuality, and chronic egg laying (i.e. the laying of eggs excessively and continuously beyond what is considered normal for their species; Schmid *et al.* 2006). Behaviours such as chronic egg laying can have detrimental effects on birds’ physical health, including depletion of calcium reserves and increased risk of becoming egg bound, which may result in potential reproductive complications or even death (Scagnelli & Tully 2017).

### 7. Lack of a ‘life plan’ for birds (e.g. end-of-life planning impacting decisions for euthanasia or rehoming + availability of qualified parrot rescues, dealing with unwanted birds)

Long lifespan of many parrot species, coupled with their challenging husbandry requirements, can result in parrots moving between multiple homes throughout their lifetime (Young *et al.* 2011; Grant *et al.* 2017). Consequently, participants identified a ‘lack of a ‘life plan” by owners for their parrots as a welfare concern. Anderson (2003) found that only 16% of owners indicated their birds were included in their formal wills; 44% had informal plans with family and friends in the event they could no longer care for their parrot, with parrots commonly relinquished following death of their primary owner/carer.

Hoppes and Gray (2010) suggested “*large parrots often lose their homes because their owner is woefully ignorant of what it takes to adopt a parrot*”, that parrots “*often scream, destroy their own feathers, bite, attack the partners of their caretakers, create a huge mess around their cage, and destroy property*”, and “*require a huge commitment in time and money*”. Similarly, Tygeson and Forkman (2023) found that excessive chirping or whistling by parrots was the largest issue of owner concern and that owners with a poor relationship with their parrot were more likely to abandon or relinquish their birds. Our participants, however, noted most of these issues as being predictable and able to be planned for or mitigated through the education of owners.

Participants also shared concerns that some rescue organisations may be ill-equipped to handle large numbers of unwanted parrots. Overstocking is becoming a significant welfare concern for rescue centres, primarily as the goal of most shelters is to provide short-term, temporary housing for birds until an appropriate home can be found (Miller & Zawistowski 2013). Possible solutions in addressing rehoming issues include improved owner education and support, as well as breeding bans to reduce the number of new individuals coming into the trade (Peng & Broom 2021; Baukhagen & Engell 2022).

### 8. Abnormal behaviours, abnormal repetitive behaviours and stereotypic behaviour

Captivity often denies parrots the opportunity to fully engage in behaviours observed in the wild, with constraints placed upon natural behaviours, including social interactions, flight, foraging and maintenance behaviours such as bathing and preening (Greenwell & Montrose 2017). The consequence of these restrictions can be associated with the expression of apparently abnormal and/or repetitive activities such as stereotypies (Mellor *et al.* 2017). In this discussion, we recognise that the term ‘abnormal’ can be challenging to consistently apply within the context of captive animal populations and interest groups differ in how they define and interpret such activities in terms of animal welfare. These perceptions differ even within academic communities focusing on this area, so a degree of caution should be exercised where different communities ascribe this term to behaviour, particularly where ‘abnormal’ is used as a proxy for ‘unnatural’ or ‘undesirable’. Nevertheless, there are behaviours that can appear functionless within the context in which they occur and may, in turn, reflect welfare concerns. The various uses of the term and their relationship with animal welfare has been considered elsewhere (e.g. Cooper & McGreevy 2002; Mason & Lathom 2004), and for our Delphi approach we did not seek to restrict our participants by imposing a definition of ‘abnormal behaviour’, but rather we sought to allow them to use the term as per the culture of their specialisms. Whilst this carried the risk of inconsistent application of the term, participants were generally consistent in those behaviours they described as abnormal, suggesting some heuristic or practical value to the term.

In parrots, the most common forms of these behaviours include feather plucking, biting, and excessive vocalisations or ‘screaming’ (Mellor *et al.* 2017). Some handlers and owners consider these behaviours to be relatively normal or adaptive, facilitating circulation or aiding digestion in the absence of other more ‘normal’ patterns of behaviour such as foraging or flying (Van Zeeland *et al.* 2009; Williams *et al.* 2017). For example, feather-damaging behaviours, which are present in an estimated 10–17% of the captive population, have been suggested as a coping strategy for negative affective states such as stress or boredom, as a consequence of living in a sub-optimal, unpredictable or uncontrollable environment (Van Zeeland *et al.* 2013; Mahdavi *et al.* 2023). There is, however, limited direct evidence of coping effects of these behaviours and further research into the development of these behaviours and consequences for the bird’s psychological state is needed. Of further note is that whilst the development of these behaviours is commonly associated with sub-standard living environments, they can become engrained and ultimately ‘detached’ from the animal’s welfare state, even when the initial factors that provoked the behaviour have since been corrected; consequently, using such behaviours as absolute welfare indicators can be fraught with issues (see Cooper *et al.* 1996; Mason & Latham 2004). Nevertheless, previous sections of this discussion have already indicated relationships between abnormal behaviours and other welfare issues, including rearing environment, limited environmental enrichment, restrictive housing, and inappropriate social grouping in parrots (Meehan *et al.* 2004; Garner *et al.* 2006).

### 9. Lack of (parrot) education to the veterinary profession

While pet owners consider and expect veterinarians to be their primary source of information regarding animal care (Coe *et al.* 2008), in this study, lack of parrot-specific education within the veterinary profession was also identified in the priority list of welfare concerns. A study of UK veterinarians by Wills and Holt (2020) identified that knowledge of and confidence in treating, diagnosing and anaesthetising exotic pet species was significantly less than for cats and dogs. Therefore, unless a veterinarian personally takes interest in avian medicine (e.g. through self-study, self-selected placements available in the final years of study, or pursuit of continual professional development [CPD]; Marino 2020), they may be underqualified to care for avian patients.

### 10. Insufficient application/enforcement of legislation and/or regulation

In the UK, animal welfare is governed by several laws, most notably the Animal Welfare Act (2006). However, the enforcement of these and other laws was deemed inadequate by our participants, and others suggest that there remains considerable scope for improved enforcement (Rudloff 2017). For example, the All-Party Parliamentary Group for Animal Welfare (All Party Parliamentary Group for Animal Welfare (APGAW) 2022) identified four barriers hindering the effective enforcement of animal welfare law including: limited resources/trained inspectors; inconsistency in enforcement; lack of training/experience among inspectors; and lack of knowledge sharing among local councils and other authorities responsible for animal protection. Compounding the issues of insufficient enforcement of broader animal welfare legislation is a lack of clear and consistent husbandry guidelines to support parrot welfare. As such, the development of evidence-based, species-specific guidelines to supplement existing legislation and support enforcement would be beneficial.

### Final notes

Interestingly, by the end of the study diseases ranked lower in priority or were excluded entirely from priority lists (despite the representation of veterinarians in the sample), with experts suggesting that the prevalence of certain diseases in the captive parrot population has been in decline. It is worth noting however that disease testing in the pet parrot population is relatively uncommon and, as such, further research may be warranted to discern trends in disease prevalence. Geriatric care and pain assessment also emerged as areas of concern amongst the experts in the study, securing a place among the priority lists for severity, duration and prevalence, but not top ten overall.

### Study considerations

We acknowledge several limitations that should be considered in the interpretation of this study’s findings. Firstly, the purposive recruitment of parrot experts known to the research team introduced a geographical bias to the study population, as most participants were from the US and the UK. As such, the study’s outcomes are unlikely to be universally applicable to captive parrot conditions across the world and caution should be exercised before generalising the identified welfare issues to all companion and other captive parrot contexts.

Purposive sampling methods are commonly carried out in Delphi studies as they rely upon the input of knowledgeable individuals from a particular field. Purposive sampling allows researchers to select individuals who possess the relevant expertise and experience, ensuring that participants are well-qualified to provide informed opinions on the subject under investigation, a consideration which is crucial for achieving consensus (Brady 2015). Here, the expert pool was increasingly composed of veterinarians as the study progressed, suggesting that veterinary viewpoints were over-represented against perspectives from academics, scientists, and other professional roles. We also acknowledge that, like any study wherein the aims are to understand people’s perspectives and generate discussion, we expect several cognitive biases were at play. For example, during the workshop phase, there is a chance that participants without postgraduate degrees (e.g. PhD or DVM) may have felt pressured to defer to the voices of the other participants (authority or expert bias); more generally such discussions may be challenged by the pressure to conform to the group (group think) and share perspectives believed to be acceptable to others ‘at the table’ (social desirability bias) (for further discussion, see Dror *et al.* 2018). Incorporating alternative recruitment approaches, like random or stratified sampling, could have provided a more diverse representation of parrot welfare experts from various regions, potentially improving the study’s applicability to diverse parrot situations worldwide (Paré *et al.* 2013). However, the effectiveness of non-purposive recruitment methods relies on randomly selected animal welfare experts possessing a relevant knowledge base in captive parrot welfare.

An additional limitation to the study was the splitting of the online workshop into two sessions. Online engagement approaches (e.g. online focus groups, workshops) allow for larger scale participant engagement as they are scalable and do not require travel to a central location (Williams *et al.* 2012; Khodyakov *et al.* 2020), but due to participants’ varying schedules across differing time zones the decision was made to conduct two online workshops. While this was intended to enhance accessibility and increase participation numbers in the final round, it altered the dynamics of the consensus-building process. Notably, the division meant participants in the separate workshops did not interact directly, potentially leading to different perspectives and insights in each group (Barrios *et al.* 2021). One considered solution entailed hosting a third round of online workshops for each workshop group, allowing participants to thoroughly discuss the rank results determined in the previous round by each workshop group. However, given the known limitation of participant drop-out in Delphi studies (Donohoe & Needham 2009) and the diminishing number of participants, this option was deemed unfeasible. Therefore, efforts were made to address potential discrepancies between workshop sessions by providing participants of workshop 1 with feedback on the results of workshop 2 via email.

Finally, we note that although study aims were framed to generate priority welfare issues affecting the wider captive parrot population across varied contexts (e.g. in homes, rescues, and in wildlife collections), several issues prioritised by our expert pool either exclusively (e.g. owner education) or perhaps especially (e.g. lack of a ‘life plan’) pertained to parrots kept as pets. However, given the presence of several issues spanning contexts of parrot-keeping (such as abnormal behaviours, issues around specialised veterinarian training, and regulatory considerations), we hope this study’s findings are useful in the consideration of welfare issues for parrots kept in a range of captive contexts.

### Animal welfare implications

To our knowledge, this study presents the first prioritisation of parrot welfare issues using expert consensus. While we initially sought to achieve expert consensus on welfare issues facing the wider captive parrot population, the expert population in this study highlighted several issues either especially pressing or unique to the pet parrot population. Through a modified Delphi approach, parrot welfare and sector professionals identified behavioural, nutritional, and housing issues as among those most pressing to address. These concerns were often noted to be deeply interconnected (e.g. the combination of inadequate nutrition and a sedentary lifestyle contribute to the development of obesity) and, as such, require multi-layered solutions. The need for improved owner and veterinarian knowledge emerged as a primary barrier to improving parrot welfare. We suggest more work is required to address the barriers in the recruitment and training of avian-interested veterinarians, including increased attention to the development of avian-specific modules and the continuing professional development of practicing veterinarians in parrot behaviour and welfare. Similarly, we recommend increased research attention on parrot owner understanding of husbandry and welfare, so as to inform how to best design educational and supportive initiatives (e.g. design and dissemination of educational material on appropriate husbandry practices, costs associated with parrot ownership, the required long-term commitment, the welfare implications of poor husbandry and breeding practices, and the exploration of more participatory engagement strategies which work with owners to co-develop strategies to improve parrot welfare).

In many cases, best practice for care and management remains unclear (e.g. nutritional requirements for many parrot species). Based on our findings, we suggest that in addition to continued research on behavioural welfare, research priorities for parrots should focus on a better understanding of the nutritional requirements of captive parrots through studies of wild counterparts, coupled with direct research into nutritional impacts of species-specific diets, establishment of appropriate care plans for geriatric parrots, and pain assessment and management. Finally, while enforcement of existing legislation was noted as a contributing factor to parrot welfare, we note that legislative change is a slow-moving process. We therefore suggest the development of species-specific husbandry guidelines, beginning with the more widely kept species, to more rapidly support the enforcement of existing laws.

## Supporting information

Chalmers et al. supplementary material Chalmers et al. supplementary material

## Supplementary material

The supplementary material for this article can be found at http://doi.org/10.1017/awf.2024.57.
