# Supplementary material for: What are the priority welfare issues facing parrots in captivity? A modified Delphi approach to establish expert consensus
Source: Anim Welf. 2024 Nov 20;33:e54. doi: 10.1017/awf.2024.57 (PMC11589074; doi:10.1017/awf.2024.57)
Supplement: Chalmers et al. supplementary material [file S0962728624000575sup001.pdf]

Chalmers et al. Survey language

SURVEY 1

What is your current employment status?

Full time
Part time
Currently seeking employment
Retired
Semi-retired
Prefer to describe ___________
Prefer not to say

What is your occupation?

[open]

How many years of experience do you have working with parrots?

3-10 years
11-20 years
21-30 years
31+ years

The following set of questions will present you with a list of welfare issues facing captive parrots. You will be asked to rank each of these issues on a 1-6 scale based upon three criteria:

Severity - defined as the severity in which the welfare issue is likely to or commonly presents, in your opinion, where 1 = mild and 6 = debilitating

Duration – defined as the likely proportion of the animal's life affected by the welfare issue, in your opinion, where 1 = fleeting and 6 = the entire duration of the individual's life

Prevalence – defined as the proportion of the population affected by the welfare issue, in your opinion, where 1 = rare and 6 = universally present in the population

Following each question you will have the opportunity to comment, if desired, upon your reasoning for your ranking. If you are unsure, please advise "I do not know" in the comments section and move onto the next question.

Next, participants received the list of the initial 28 welfare issues (see Table 1 in paper for issues) presented on 1-6 scales in terms of severity, duration, and prevalence.

Prior to survey 1 conclusion, participants received a second set of demographic questions:

What is your age group?

18-24
25-34
35-44
45-54
55-64
65+

What is your gender?

Man
Woman
Non-binary/non-conforming
Prefer to self describe _______
Prefer not to say

What is your highest level of education?

GCSE or equivalent (e.g. schooling up to 16)
A levels of equivalent (e.g. Level 3 Diploma, High school diploma)
Vocational/trade school
Associates degree
Bachelor’s degree
Postgraduate (e.g. MS, MA, PhD)
Professional (e.g. DVM, MD, DO, JD)
Prefer not to say

What country do you live in?

[open]

UNIVERSITY OF LINCOLN

Survey 1(B) Parrot Welfare: A Modified Delphi Study to Determine Priority Welfare Issues in Captive Parrots (Psittaciformes)

Page 1: Survey Introduction

Title of Study: Parrot Welfare: A Modified Delphi Study to Determine Priority Welfare Issues in Captive Parrots (Psittaciformes)

You are asked to participate in a research study conducted by *Rhianne Chalmers* from the *Department of Life Sciences* at the *University of Lincoln* and supervised by Drs. Jonathan Cooper and Beth Ventura. The results of this project will contribute to the fulfilment of a Masters by Research thesis for the successful completion of the *Masters by Research in Animal Behaviour and Welfare.*

If you have any questions or concerns regarding the research please contact:

Rhianne Chalmers

Masters Student, Department of Life Sciences, University of Lincoln

27110083@students.lincoln.ac.uk

Purpose of the Study

This is a modified Delphi study consisting of up to three stages: two survey rounds and one online workshop. This is the **first survey round section B. Section B has been added to the study in order to clarify certain welfare issues and to rank new welfare**

issues identified by participants. The objective is to create a consensus on the priority welfare issues affecting captive psittacines today. The welfare issues identified as most important will allow for targeted research and education which is essential to improve the welfare of parrots in captivity.

For the purpose of this study “parrot(s)” will refer to all species belonging to the order Psittaciformes.

Confidentiality

Every effort will be made to ensure confidentiality of any identifying information that is obtained for the purpose of the study. As this study involves multiple stages, email addresses will be collected at each stage to allow later linking of responses across stages; once data from each round has been collected, email addresses will be removed from responses. Upon completion of data collection all data will be anonymised for publication. Data from the survey will be stored in password protected files on the University of Lincoln server and will only be accessible by the research team.

Data will be retained for up to five years after the results of the research are presented.

Please note if you participate in stage 3 (online workshop), you will participate in a live discussion with other individuals and will not be anonymous during this stage. However, all participants will be requested to keep any identifiable information of participants confidential.

Rights of Participants

You have the right to withdraw your participation from the study and consent to participate at any stage and withdraw any or all responses given. Choosing not to take part or withdrawing from the study part way will not disadvantage you in any way.

All research conducted by the University of Lincoln is looked at by an independent group of people, called a Research Ethics Committee, to protect your rights, dignity and wellbeing. This study has been reviewed and given favourable opinion by a University of Lincoln Research Ethics Committee [project ID 11911].

Please consent below ✱ *Required*

- [ ] I confirm I have been presented with and have read the Participants Information Sheet
- [ ] I confirm I am at least 18 years of age and have at least 3 years professional experience involving parrots
- [ ] I consent to participate in this study

Please insert your email address (Please use the same email address used in the previous survey). ✱ *Required*

Page 2: Re-Ranking Welfare Issues

The following set of questions will present you with **welfare issues that required clarification based upon participant feedback**. You will be asked to re-rank each of these issues on a 1-6 scale based upon three criteria:

Severity - defined as the severity in which the welfare issue is likely to or commonly presents, in your opinion, where 1 = mild and 6 = debilitating

Duration – defined as the likely proportion of the animal's life affected by the welfare issue, in your opinion, where 1 = fleeting and 6 = the entire duration of the individual's life

Prevalence – defined as the proportion of the population affected by the welfare issue, in your opinion, where 1 = rare and 6 = universally present in the population

Following each question you will have the opportunity to comment, if desired, upon your reasoning for your ranking. If you are unsure, please advise “I do not know” in the comments section and move onto the next question.

The hand-rearing of parrot chicks (to include the selling of un-weaned birds to inexperienced owners/handlers for the purpose of hand-rearing)

Please don't select more than 1 answer(s) per row.

	1	2	3	4	5	6
Severity	☐	☐	☐	☐	☐	☐
Duration	☐	☐	☐	☐	☐	☐
Prevalence	☐	☐	☐	☐	☐	☐

Comments:

Unsuitable cage/aviary/enclosure location (resulting in sleep deprivation, hypervigilance, physical harm)

Please don't select more than 1 answer(s) per row.

	1	2	3	4	5	6
Severity	☐	☐	☐	☐	☐	☐
Duration	☐	☐	☐	☐	☐	☐
Prevalence	☐	☐	☐	☐	☐	☐

Comments:

Inadequate cage/aviary/enclosure size (restricting movement, flight ability, foraging and other natural behaviours)

Please don't select more than 1 answer(s) per row.

	1	2	3	4	5	6
Severity	☐	☐	☐	☐	☐	☐
Duration	☐	☐	☐	☐	☐	☐
Prevalence	☐	☐	☐	☐	☐	☐

Comments:

Wing clipping resulting in physical harm and restricted flight

Please don't select more than 1 answer(s) per row.

	1	2	3	4	5	6
Severity	☐	☐	☐	☐	☐	☐
Duration	☐	☐	☐	☐	☐	☐
Prevalence	☐	☐	☐	☐	☐	☐

Comments:

Page 3: Re-Ranking of Welfare Issues

The following set of questions will present you with **new welfare issues identified by participants in survey one**. You will be asked to rank each of these issues on a 1-6 scale based upon three criteria:

Severity - defined as the severity in which the welfare issue is likely to or commonly presents, in your opinion, where 1 = mild and 6 = debilitating

Duration – defined as the likely proportion of the animal's life affected by the welfare issue, in your opinion, where 1 = fleeting and 6 = the entire duration of the individual's life

Prevalence – defined as the proportion of the population affected by the welfare issue, in your opinion, where 1 = rare and 6 = universally present in the population

Following each question you will have the opportunity to comment, if desired, upon your reasoning for your ranking. If you are unsure, please advise "I do not know" in the comments section and move onto the next question.

Enforced prolonged periods of complete darkness as a result of cages being covered and/or kept in rooms with no light regulation/ no transitionary period.

Please don't select more than 1 answer(s) per row.

	1	2	3	4	5	6
Severity	☐	☐	☐	☐	☐	☐
Duration	☐	☐	☐	☐	☐	☐
Prevalence	☐	☐	☐	☐	☐	☐

Comments:

Lack of environmental enrichment resulting in a cognitively unchallenging environment

Please don't select more than 1 answer(s) per row.

	1	2	3	4	5	6
Severity	☐	☐	☐	☐	☐	☐
Duration	☐	☐	☐	☐	☐	☐
Prevalence	☐	☐	☐	☐	☐	☐

Comments:

Improper breeding management, e.g. genetic testing, disease testing, husbandry management

Please don't select more than 1 answer(s) per row.

	1	2	3	4	5	6
Severity	☐	☐	☐	☐	☐	☐
Duration	☐	☐	☐	☐	☐	☐
Prevalence	☐	☐	☐	☐	☐	☐

Comments:

Inability to carry out mating behaviours

Please don't select more than 1 answer(s) per row.

	1	2	3	4	5	6
Severity	☐	☐	☐	☐	☐	☐
Duration	☐	☐	☐	☐	☐	☐
Prevalence	☐	☐	☐	☐	☐	☐

Comments:

Inadequate housing temperature

Please don't select more than 1 answer(s) per row.

	1	2	3	4	5	6
Severity	☐	☐	☐	☐	☐	☐
Duration	☐	☐	☐	☐	☐	☐
Prevalence	☐	☐	☐	☐	☐	☐

Comments:

Inappropriate and/or unsafe toys (i.e. improper materials, potential physical harm from damaged toys)

Please don't select more than 1 answer(s) per row.

	1	2	3	4	5	6
Severity	☐	☐	☐	☐	☐	☐
Duration	☐	☐	☐	☐	☐	☐
Prevalence	☐	☐	☐	☐	☐	☐

Comments:

Insufficient legislation and/or regulaton surrounding the sale of parrots in commercial settings e.g. pet stores, auction houses and shows resulting in unregulated welfare practices.

Please don't select more than 1 answer(s) per row.

	1	2	3	4	5	6
Severity	☐	☐	☐	☐	☐	☐
Duration	☐	☐	☐	☐	☐	☐
Prevalence	☐	☐	☐	☐	☐	☐

Comments:

Non-avian vets treating avian (parrot) species, often resulting in misdiagnosis or improper care

Please don't select more than 1 answer(s) per row.

	1	2	3	4	5	6
Severity	☐	☐	☐	☐	☐	☐
Duration	☐	☐	☐	☐	☐	☐
Prevalence	☐	☐	☐	☐	☐	☐

Comments:

Lack of shelters or rescues with the ability, capacity or knowledge to take in and re-home birds to suitable homes for the duration of the birds' lives

Please don't select more than 1 answer(s) per row.

	1	2	3	4	5	6
Severity	☐	☐	☐	☐	☐	☐
Duration	☐	☐	☐	☐	☐	☐
Prevalence	☐	☐	☐	☐	☐	☐

Comments:

Page 4: Final page

You have reached the end of the survey.

Thank you for participating in survey one (B) of the modified Delphi study to identify priority welfare issues in captive parrots (Psittaciformes).

If you have any questions regarding the survey you have just completed please contact the researcher by emailing 27110083@students.lincoln.ac.uk.

You will be contacted within the next 2 weeks of the survey closing date with an invitation to take part in stage two of the modified Delphi study which consists of an online workshop. Your participation in stage three would be entirely voluntary. Thank you again!

Survey 2 Parrot Welfare: A Modified Delphi Study to Determine Priority Welfare Issues in Captive Parrots (Psittaciformes) (copy) (copy)

Page 1: Survey Introduction

Title of Study: Parrot Welfare: A Modified Delphi Study to Determine Priority Welfare Issues in Captive Parrots (Psittaciformes)

You are asked to participate in a research study conducted by *Rhianne Chalmers* from the *Department of Life Sciences* at the *University of Lincoln* and supervised by Drs. Jonathan Cooper and Beth Ventura. The results of this project will contribute to the fulfilment of a Masters by Research thesis for the successful completion of the *Masters by Research in Animal Behaviour and Welfare.*

If you have any questions or concerns regarding the research please contact:

Rhianne Chalmers

Masters Student, Department of Life Sciences, University of Lincoln

27110083@students.lincoln.ac.uk

Purpose of the Study

This is a modified Delphi study consisting of up to three stages: two survey rounds and one online workshop. This is the **second survey round.** The objective is to create a consensus on the priority welfare issues affecting captive psittacines today. The welfare issues identified as most important will allow for targeted research and education which is essential to improve the welfare of parrots in captivity.

For the purpose of this study “parrot(s)” will refer to all species belonging to the order Psittaciformes.

Potential Benefits of the Study

The research will provide valuable insight into the current welfare issues affecting captive parrots.

Confidentiality

Every effort will be made to ensure confidentiality of any identifying information that is obtained for the purpose of the study. As this study involves multiple stages, email addresses will be collected at each stage to allow later linking of responses across stages; once data from each round has been collected, email addresses will be removed from responses. Upon completion of data collection all data will be anonymised for publication. Data from the survey will be stored in password protected files on the University of Lincoln server and will only be accessible by the research team.

Data will be retained for up to five years after the results of the research are presented.

Please note if you participate in stage 3 (online workshop), you will participate in a live discussion with other individuals and will not be anonymous during this stage. However, all participants will be requested to keep any identifiable information of participants confidential.

Rights of Participants

You have the right to withdraw your participation from the study and consent to participate at any stage and withdraw any or all responses given. Choosing not to take part or withdrawing from the study part way will not disadvantage you in any way.

All research conducted by the University of Lincoln is looked at by an independent group of people, called a Research Ethics Committee, to protect your rights, dignity and wellbeing. This study has been reviewed and given favourable opinion by a University of Lincoln Research Ethics Committee [project ID 11911].

Participation Withdrawal

It is your choice whether you wish to participate in this study. If you chose to participate in this study you may withdraw at any time during data collection by contacting the researcher as detailed above. Subject to your wishes you may choose to partially or fully remove your responses from the study. Partially removed referring to whether you agree for the researcher to continue to use the results from one or more parts of the study.

Please note, where the data has already been anonymised and submitted for the presentation of the study, publication or use in future research, the researcher will be unable to remove your anonymised data from the analysis.

Participant Information Sheet

For further information on participation, please find the Participant Information Sheet here: **https://static.onlinesurveys.ac.uk/media/account/69/survey/1033839/question/participants_information_sheet.pdf**

1. Please consent below ✱ *Required*

- ☐ I confirm I have been presented with and have read the Participants Information Sheet
- ☐ I confirm I am at least 18 years of age and have at least 3 years professional experience involving parrots
- ☐ I consent to participate in this study

2. Please insert your email address (Please use the same email address as used in previous surveys). ✱ *Required*

Page 2: Survey 1 Results Consensus

Below you will find shortened lists of the welfare issues from Survey Round 1, arranged according to their average ranking from highest to lowest in terms of *severity*, *duration*, and *prevalence*. The average ranking will be included in parentheses after each welfare issue.

Please now indicate whether you agree or disagree with the relative position of each welfare issue, by selecting either:

Agree- meaning that you agree with the relative position of the issue on that list

Disagree-should be lower- meaning that you believe the issue should appear lower in the rankings on that list

Disagree-should be higher- meaning that you believe the issue should appear higher in the rankings on that list

3. Severity (defined as the severity in which the welfare issue is likely to or commonly presents, in your opinion) ✱
Required

Please don't select more than 1 answer(s) per row.

Please select exactly 24 answer(s).

	Agree	Disagree-should be lower	Disagree-should be higher	
1	☐	☐	☐	Lack of environmental enrichment resulting in a cognitively unchallenging environment (5.17/6)
2	☐	☐	☐	Insufficient legislation and/or regulation surrounding the sale of parrots in commercial settings e.g. pet stores, auction houses and shows resulting in unregulated welfare practices (5.08/6)
3	☐	☐	☐	Inadequate cage/aviary/enclosure size (restricting movement, flight ability, foraging and other natural behaviours) (5.08/6)
4	☐	☐	☐	Psittacine Beak and Feather Disease (PBFD) (a viral disease belonging to the genus Circovirus, causing progressive feather, claw and beak malformation and necrosis) (4.78/6)
5	☐	☐	☐	Social isolation from conspecifics (i.e. other parrots) (4.77/6)
6	☐	☐	☐	Proventricular Dilation Disease (PDD)/Avian Bornaviral Ganglioneuritis (ABG) caused by Avian Bornavirus (ABV) (an inflammatory disease characterized by proventricular dilation and blockage of the passage of digesta) (4.73/6)

7				Parrot owner/carer unwillingness or inability to seek and/or implement veterinary and/or behavioural advice (4.62/6)
8				Abnormal behaviours (ABs), abnormal repetitive behaviours (ARBs) and stereotypic behaviour (4.58/6)
9				The hand-rearing of parrot chicks (to include the selling of un-weaned birds to inexperienced owners/handlers for the purpose of hand-rearing) (4.5/6)
10				Unsuitable cage/aviary/enclosure location (resulting in sleep deprivation, hypervigilance, physical harm) (4.5/6)
11				Wing clipping resulting in physical harm and restricted flight (4.5/6)
12				Avian polyomavirus (APV), previously referred to as Budgerigar Fledgling Disease (BFD) (an inflammatory virus affecting juveniles in several parrot species) (4.45/6)
13				Aspergillosis (a respiratory fungal disease caused primarily by fungal organisms of the genus Aspergillus and to a lesser extent with fungi of other genera such a Penicillium and Mucor) (4.44/6)
14				Non-avian vets treating avian (parrot) species, often resulting in misdiagnosis or improper care (4.25/6)
15				Limited to no foraging opportunities (4.24/6)
16				Access and/or exposure to toxic foods, plants, chemicals and other harmful objects (4.24/6)
17				Unbalanced or nutritionally-deficient diet (4.19/6)
18				Overbonding with owner/carer(s) (4.19/6)
19				The promotion of seed and other incomplete diets as 'whole' diets (4.17/6)
20				Insufficient light exposure and/or no provision of UV lighting (4.16/6)
21				Improper breeding management, e.g. genetic testing, disease testing, husbandry management (4.16/6)
22				Lack of species-specific dietary requirements (4.08/6)
23				Inadequate housing temperature (4/6)

24	☐	☐	☐	Lack of shelters or rescues with the ability, capacity or knowledge to take in and re-home birds to suitable homes for the duration of the birds' lives (4/6)

3.a. If desired, please add any commentary to your agreement/disagreement choices:

4. Duration (defined as the likely proportion of the animal's life affected by the welfare issue, in your opinion) 1 = Agree
2 = Disagree- should be lower
3 = Disagree- should be higher ✱ *Required*

Please don't select more than 1 answer(s) per row.

Please select exactly 27 answer(s).

	1	2	3	
1	☐	☐	☐	Inadequate cage/aviary/enclosure size (restricting movement, flight ability, foraging and other natural behaviours) (5.67/6)
2	☐	☐	☐	Unsuitable cage/aviary/enclosure location (resulting in sleep deprivation, hypervigilance, physical harm) (5.5/6)
3	☐	☐	☐	Lack of environmental enrichment resulting in a cognitively unchallenging environment (5.5/6)
4	☐	☐	☐	Social isolation from conspecifics (i.e. other parrots) (5.35/6)
5	☐	☐	☐	The hand-rearing of parrot chicks (to include the selling of un-weaned birds to inexperienced owners/handlers for the purpose of hand-rearing) (5.17/6)
6	☐	☐	☐	Improper breeding management, e.g. genetic testing, disease testing, husbandry management (5.17/6)
7	☐	☐	☐	Unbalanced or nutritionally-deficient diet (5.12/6)
8	☐	☐	☐	Lack of species-specific dietary requirements (5.04/6)
9	☐	☐	☐	Limited to no foraging opportunities (5/6)
10	☐	☐	☐	Insufficient light exposure and/or no provision of UV lighting (5/6)

11	☐	☐	☐	Abnormal behaviours (ABs), abnormal repetitive behaviours (ARBs) and stereotypic behaviour (5/6)
12	☐	☐	☐	Overbonding with owner/carer(s) (5/6)
13	☐	☐	☐	Parrot owner/carer unwillingness or inability to seek and/or implement veterinary and/or behavioural advice (4.96/6)
14	☐	☐	☐	Insufficient legislation and/or regulation surrounding the sale of parrots in commercial settings e.g. pet stores, auction houses and shows resulting in unregulated welfare practices (4.92/6)
15	☐	☐	☐	Psittacine Beak and Feather Disease (PBFD) (a viral disease belonging to the genus Circovirus, causing progressive feather, claw and beak malformation and necrosis) (4.91/6)
16	☐	☐	☐	The promotion of seed and other incomplete diets as 'whole' diets (4.79/6)
17	☐	☐	☐	Inadequate perches (e.g. with respect to number, size, material, variety, and/or location) (4.77/6)
18	☐	☐	☐	Unsanitary cage/aviary/enclosure conditions (e.g. resulting from improper cleaning and environmental [e.g. ventilation, humidity] control) (4.69/6)
19	☐	☐	☐	Proventricular Dilation Disease (PDD)/Avian Bornaviral Ganglioneuritis (ABG) caused by Avian Bornavirus (ABV) (an inflammatory disease characterized by proventricular dilation and blockage of the passage of digesta) (4.61/6)
20	☐	☐	☐	Wing clipping resulting in physical harm and restricted flight (4.58/6)
21	☐	☐	☐	Lack of shelters or rescues with the ability, capacity or knowledge to take in and re-home birds to suitable homes for the duration of the birds' lives (4.58/6)
22	☐	☐	☐	Inability to carry out mating behaviours (4.42/6)
23	☐	☐	☐	Non-avian vets treating avian (parrot) species, often resulting in misdiagnosis or improper care (4.42/6)
24	☐	☐	☐	Absence of predictability, routine and control (4.32/6)
25	☐	☐	☐	Inadequate housing temperature (4.08/6)
26	☐	☐	☐	Incompatible social groups (4.04/6)

27	☐	☐	☐	Enforced prolonged periods of complete darkness as a result of cages being covered and/or kept in rooms with no light regulation/ no transitionary period (4/6)

4.a. If desired, please add any commentary to your agreement/disagreement choices:

5. Prevalence (defined as the proportion of the population affected by the welfare issue, in your opinion) Where: 1 = Agree
2 = Disagree- should be lower
3 = Disagree- should be higher ✱ *Required*

Please don't select more than 1 answer(s) per row.

Please select exactly 16 answer(s).

	1	2	3	
1	☐	☐	☐	Inadequate cage/aviary/enclosure size (restricting movement, flight ability, foraging and other natural behaviours) (5.08/6)
2	☐	☐	☐	Insufficient legislation and/or regulation surrounding the sale of parrots in commercial settings e.g. pet stores, auction houses and shows resulting in unregulated welfare practices (4.83/6)
3	☐	☐	☐	Lack of environmental enrichment resulting in a cognitively unchallenging environment (4.75/6)
4	☐	☐	☐	Inability to carry out mating behaviours (4.67/6)
5	☐	☐	☐	Social isolation from conspecifics (i.e. other parrots) (4.65/6)
6	☐	☐	☐	Limited to no foraging opportunities (4.56/6)
7	☐	☐	☐	Lack of species-specific dietary requirements (4.54/6)
8	☐	☐	☐	Unbalanced or nutritionally-deficient diet (4.5/6)
9	☐	☐	☐	Insufficient light exposure and/or no provision of UV lighting (4.44/6)
10	☐	☐	☐	Unsuitable cage/aviary/enclosure location (resulting in sleep deprivation, hypervigilance, physical harm) (4.33/6)

11	☐	☐	☐	The promotion of seed and other incomplete diets as 'whole' diets (4.21/6)
12	☐	☐	☐	Improper breeding management, e.g. genetic testing, disease testing, husbandry management (4.17/6)
13	☐	☐	☐	Lack of shelters or rescues with the ability, capacity or knowledge to take in and re-home birds to suitable homes for the duration of the birds' lives (4.17/6)
14	☐	☐	☐	Abnormal behaviours (ABs), abnormal repetitive behaviours (ARBs) and stereotypic behaviour (4.12/6)
15	☐	☐	☐	Parrot owner/carer unwillingness or inability to seek and/or implement veterinary and/or behavioural advice (4.04/6)
16	☐	☐	☐	Non-avian vets treating avian (parrot) species, often resulting in misdiagnosis or improper care (4/6)

5.a. If desired, please add any commentary to your agreement/disagreement choices:

Page 3: Final page

You have reached the end of the survey.

Thank you for participating in stage two of the modified Delphi study to identify priority welfare issues in captive parrots (Psittaciformes).

If you have any questions regarding the survey you have just completed please contact the researcher by emailing 27110083@students.lincoln.ac.uk.

You will be contacted within the next 2-4 weeks of the survey closing date with an invitation to take part in the final stage of the modified Delphi study, which consists of an online workshop. Your participation in stage three would be entirely voluntary. Thank you again!
